# Autobiographical Meaning Making Protects the Sense of Self-Continuity Past Forced Migration

**DOI:** 10.3389/fpsyg.2021.618343

**Published:** 2021-02-25

**Authors:** Christin Camia, Rida Zafar

**Affiliations:** ^1^Department of Psychology, Zayed University, Abu Dhabi, United Arab Emirates; ^2^Psychology Program, New York University Abu Dhabi, Abu Dhabi, United Arab Emirates

**Keywords:** autobiographical reasoning, life narrative, personal identity, meaning making, well-being

## Abstract

Forced migration changes people’s lives and their sense of self-continuity fundamentally. One memory-based mechanism to protect the sense of self-continuity and psychological well-being is autobiographical meaning making, enabling individuals to explain change in personality and life by connecting personal experiences and other distant parts of life to the self and its development. Aiming to replicate and extend prior research, the current study investigated whether autobiographical meaning making has the potential to support the sense of self-continuity in refugees. We therefore collected life narratives from 31 refugees that were coded for autobiographical reasoning, self-event connections, and global narrative coherence. In line with prior research, results suggest that autobiographical meaning making relates to a higher sense of self-continuity and less psychological distress. Yet, if refugees experienced many continuing postdisplacement stressors in addition to their forced displacement, autobiographical meaning making was associated with higher self-discontinuity and greater psychological distress, especially with trauma-related symptoms such as memory intrusion and hyperarousal. Altogether, results indicate that autobiographical meaning making helps to compensate the effects of extreme biographical disruptions on the sense of self-continuity, as long as the stress caused by the biographical change is not overwhelming or too protracted.

## Introduction

Forced migration is an ongoing challenge for societies in many regions of the world. According to the United Nations High Commissioner for Refugees United Nations High Commissioner for Refugees [UNHCR] (2020), 79.5 million people, i.e., about 1% of the world population, were forcibly displaced at the end of 2019. Becoming a refugee instigates numerous psychological problems (Ullmann et al., 2015), as forced migration is mainly a process of losses. Individuals who are forced to leave their home country lose their homeland, their community, their family and friends, their language, their culture, and their physical safety (Carta et al., 2005). Thus, forced migration might be one of the greatest biographical disruptions people can face. Amid these drastic changes, refugees are challenged to preserve or regain their sense of self-continuity, protecting their psychological well-being. Inspired by prior research showing that autobiographical meaning making buffers the impact of negative life events on the sense of self-continuity (Habermas and Köber, 2015a), the current study investigated whether autobiographical meaning making has the potential to support the sense of self-continuity in extreme circumstances such as forced migration.

## The Sense of Self-Continuity and Autobiographical Memory

The pre-reflective sense of self-continuity provides a person with the feeling to be the same and continuous over time (Erikson, 1968). A disturbed sense of self-continuity is the feeling that an individual cannot relate to his or her former self anymore, sometimes perceived as estrangement from, and lacking sense of ownership of one’s body, thoughts, and feelings. While a disturbed sense of self-continuity can be a chronic symptom in personality disorders (Kernberg, 1984), healthy personalities can also temporarily experience a disturbed sense of self-continuity in times of change and stress, which can be challenging for the identity (Habermas and Köber, 2015b; Jiang et al., 2020). In such times, individuals are motivated to repair and regain their sense of self-continuity (Erikson, 1968) and often do so by employing their autobiographical memories (Bluck, 2003; Bluck and Liao, 2013). Research on autobiographical memory suggests two main mechanisms to support the sense of self-continuity: remembering the past and consciously reflecting the past (Bluck and Liao, 2013).

Remembering the past is to immerse in nostalgic memories, creating an emotional, sometimes wistful, connection between the past and the remembering individual (Sedikides et al., 2008; Prebble et al., 2013). Such remembering relies on the discrete recall of specific events, images, sensations, or feelings of one’s past and does not involve conscious reflection of one’s life story. As voluntary or involuntary memories come to mind, they provide the self with the sense of existing over time and thus provide a basic sense of chronological self-continuity (Bluck and Liao, 2013). A recent study showed that nostalgic remembering increases the sense of self-continuity in refugees, especially in those with higher levels of resilience (Wildschut et al., 2019). If, however, the contrast between present and past life is so stark that self-continuity is profoundly disturbed, nostalgic remembering falls short in repairing the sense of self-continuity as it tends to intensify the awareness of the cherished but irrecoverable past and thus damages well-being (Iyer and Jetten, 2011).

Profound disturbances of the sense of self-continuity result from tragic negative events such as a sudden loss of close others (Bauer and Bonanno, 2001), body parts or substantial health (Hinojosa et al., 2008; Uzer and Brown, 2015), or major roles due to job loss or retirement (Nuttman-Shwartz, 2008). Because such drastic changes put past and present life in stark contrast to each other, they might be labeled *biographical disruptions* or *biographical ruptures*. Forced displacement certainly counts as biographical disruption as it entails profound changes and losses (Carta et al., 2005). Hence, refugees and migrants perceive a sharp dissimilarity between their past and present life (Shi and Brown, 2016) and show, discontinuous sense of self when remembering their past (Gemignani, 2011; Oguz, 2012; Puvimanasinghe et al., 2015; Witney and Bates, 2016).

In times of biographical disruptions, it needs the conscious reflection of the past to reestablish a retrospective sense of self-continuity (Bluck and Liao, 2013). The conscious reflection of the past is the active effort to create a coherent life story and to engage in effortful autobiographical meaning making in order to explain and negotiate personal changes (Ricoeur, 1992; Bluck and Liao, 2013; Habermas and Köber, 2015b). Going beyond mere remembering of the past, autobiographical meaning making requires the deliberate reflection of past life events and to extract personal meaning from past experiences in order to yield a coherent account of identity in time (Staudinger, 2001; D’Argembeau et al., 2014; Lavallee et al., 2019). Because this study focuses on the conscious reflection of the past, autobiographical meaning making and its effects on self-continuity and psychological distress are described in greater detail below.

## Autobiographical Meaning Making Relates to Self-Continuity and Psychological Distress

In life narratives covering the entire life span, many different life events are expected to be included and to be related to other parts of life and to the narrator’s personality development up to the present. Therefore, life narratives require global narrative coherence in order to convey personal identity in a comprehensible manner. Overarching global coherence of life narratives has been conceptualized in three major kinds of textual coherence: temporal, causal–motivational, and thematic coherence (Habermas and Bluck, 2000). Global temporal coherence refers to the temporal organization of events and the elaboration of beginnings and endings (Köber and Habermas, 2017). Global causal–motivational coherence captures the narrator’s explanations of causes, motivations, and consequences of past events and actions for the personal life course and development. Global thematic coherence concerns dominant life themes, which might emerge throughout the narrative as different parts of life and of the narrator’s personality match in similarity of themes (Köber et al., 2015).

Within a life narrative, global narrative coherence results from linking events to the narrator’s personality, portraying how one event caused other events or how motives led to actions producing consequences beyond the boundaries of local events. This conscious reflection on life events from a broader biographical perspective is termed *autobiographical reasoning*, which involves the use of *autobiographical arguments*. Autobiographical arguments explain change and stability in personality and life by connecting personal experiences and other distant parts of life to the self and its development (Habermas, 2011). Hence, autobiographical reasoning is a powerful memory-based mechanism to create a coherent life story and a coherent sense of self because it explains both enduring change and stability in the self by reference to life events (Köber et al., 2015).

One specific form of autobiographical arguments is *self-event connections*, which are defined as explicit statements that link personal experiences to an enduring aspect of self (Pasupathi et al., 2007). Through self-event connections, past life events can be related to personality dispositions, current values, current perspectives about the world or self in general, personal growth, and important relationships informing the sense of self (Merrill et al., 2016). Thus, while other forms of autobiographical arguments rather explain enduring change and personality development, self-event connections tend to emphasize enduring aspects of the self. In contrast to other autobiographical arguments, which are mainly explanatory, self-event connections can be positive or negative in their content as individuals evaluate life events and the impact certain life events may have had on enduring aspects of their self. Positive self-event connections appreciate the past or the current self, whereas negative self-event connections disapprove the past or the current self (Merrill et al., 2016).

Autobiographical meaning making, that is, crafting coherent autobiographical accounts, autobiographical reasoning, and creating self-event connections, has been found to be protective for the sense of personal identity and psychological well-being amid and past negative life events. In a study with Belgian undergraduate students, narrative coherence appeared to safeguard against the psychological distress caused by academic failure, at least in the short term (Vanderveren et al., 2020). Furthermore, in a German non-clinical sample, autobiographical reasoning has been found to buffer the impairing effect of negative events on the sense of self-continuity. This effect, however, was only observed in individuals who had experienced biographical disruptions such as relocation, illness, or death prior to the study (Habermas and Köber, 2015a). Apparently, the sense of self-continuity is reduced only in times of biographical ruptures, but can be reestablished by conscious autobiographical meaning making. Similarly, positive self-event connections seem to shield against psychological distress and to strengthen the sense of identity when processing negative or traumatic life events in personal narratives. Negative self-event connections, in contrast, impair the sense of identity and psychological well-being (Banks and Salmon, 2013; Merrill et al., 2016).

Seeking to replicate and extend these prior results, this study investigated whether autobiographical meaning making compensates for the adverse effects of forced migration on the sense of self-continuity (hypothesis 1) and psychological distress (hypothesis 2). We hypothesized that this compensating effect would show as negative correlations of the sense of self-discontinuity with narrative coherence (hypothesis 1a), autobiographical reasoning (hypothesis 1b), and positive self-event connections (hypothesis 1c). Similarly, we hypothesized that the level of psychological distress correlates negatively with narrative coherence (hypothesis 2a), autobiographical reasoning (hypothesis 2b), and positive self-event connections (hypothesis 2c).

## Materials and Methods

### Participants

In total, 32 individuals were recruited to participate in this study. However, the data collection of one participant failed due to technical problems. Therefore, the sample presented here consists in total of 31 participants, including one female. Data collection took place in Germany between May and July 2015 when the European “refugee crisis” was underway (Triandafyllidou, 2018). Participants ranged in age from 19 to 58 years (*M* = 31.39; *SD* = 9.91) and came mostly from countries such as Eritrea, Ghana, Afghanistan, Ethiopia, Togo, and Syria. Almost half of the sample (48.40%) had finished high school, 29.00% had completed university, and the remaining 22.60% had elementary or no school education. Main reasons for fleeing their country were war, forced military service, poverty, and political or religious persecution. On average, refugees had been in Germany for 18.45 months (range 8–43 months). About half of the participants (*N* = 16) were accommodated in a refugee camp in Germany, while the 15 remaining participants stayed in apartments or in church asylum in a bigger German city. Several of those who were not living in institutional accommodation were at times homeless before obtaining other forms of accommodation. Although participants were officially recognized as refugees by the European Union (38.70%) or by Germany (61.30%), the majority (61.30%) had not obtained the right for unlimited residence in Germany, keeping them in uncertainty about their future.

### Procedure

Participants were recruited with the help of a research assistant, who volunteered in a refugee camp, and by word of mouth among the refugee community. This study was approved by the Research Ethics Committee of Fachbereich 05 Psychologie und Sportwissenschaften of the Department of Psychology, Goethe University Frankfurt (#2015-127) on March 17, 2015.

Following consent, participants first told their life narrative and then completed several questionnaires. All interviews were conducted in English and took place in the university or in a silent room in the refugee camp. Interview sessions lasted between 1 and 1.5 h for which participants were compensated with 20 Euros.

### Materials

#### Life Narratives

Before providing the life narrative, participants named their seven most important memories and brought them in chronological order. This served to familiarize participants with the concept of telling their life story and ensured that life narratives comprised specific events. Participants were asked to narrate their life for about 25 min without being interrupted. They were instructed to include the seven most important memories and to tell their life such as to explain how they had become the person they were at present. Interviewers only encouraged to continue but asked no questions.

#### Questionnaires

Following the narration of their lives, participants completed several questionnaires. We measured the general psychological distress with the Brief Symptom Inventory (Derogatis and Melisaratos, 1983), the specific psychological distress related to the flight from the home country with the revised Impact of Event Scale (IES-R, Weiss, 2007), the objective change in life that took place since their flight, and their sense of self-discontinuity.

#### The Brief Symptom Inventory

The Brief Symptom Inventory (Derogatis and Melisaratos, 1983) measures a range of psychological symptomatology such as somatization, depression, hostility, anxiety, paranoia, phobias, and obsessive compulsive tendencies. Combining the numbers of psychological symptoms and the intensity of the perceived stress, the Global Severity Index measures the general distress and is thus the single best indicator of distress levels currently experienced by the individual. Internal consistency was high, Cronbach’s α = 0.91.

#### The Impact of Event Scale

The revised version of the Impact of Event Scale (IES-R; Weiss, 2007) was used to measure participants’ traumatic stress in relation to the experience of the flight. Three subscales measure intrusion, avoidance, and hyperarousal, which are typical symptomatic responses following exposure to traumatic life events. Intrusion is characterized by nightmares or involuntary memory flashbacks; avoidance is the more or less conscious effort to not think or talk about the traumatic event; and hyperarousal is described by anger, irritability, trouble concentrating, and hypervigilance. We used the IES-R to measure on a 5-point Likert scale (0 = not at all to 4 = extremely) the intensity with which participants experienced in the last 7 days such symptoms with specific respect to their flight. Internal consistency was high, Cronbach’s α = 0.89.

#### Objective Change in Life

Using the scale employed by Habermas and Köber (2015a), we measured how much objective life circumstances had changed in the participants’ lives since they left their country. Thus, this scale assessed continuous stressors that protract the initial stress caused by the flight. Given the life circumstance of the participants, we slightly adapted the scale and asked for absolute frequencies of disruptive life events following their flight: loss of or separation from partner, beginning of new love relationship, loss or gain of friends, severe illness or death in close persons, and moving residencies within Germany. The scale ranged from 1 (never) and 2 (once) to 5 (four times) and 6 (more than four times). Due to the heterogeneity of items, internal consistency was moderate, Cronbach’s α = 0.64. Because events differed in severity and frequency, we z-standardized each item before averaging them.

#### Sense of Self-Discontinuity

Following Habermas and Köber (2015a), we measured the inversed sense of self-continuity, that of self-discontinuity. Participants reflected their feeling of familiarity with themselves in the past when they were still living in their home country. The four items were “I can still relate to the kind of person I was and to how I felt before I left my country” (inverted); “When I think back to how I was before I left my country, it feels a little unfamiliar”; “When I look at pictures of myself before I left my country, it feels a little unfamiliar”; and “I have the feeling that at the core I am the same person I was before I left my country” (inverted). Responses were scaled from 1 “not true at all” to 6 “absolutely true.” Internal consistency was good with Cronbach’s α = 0.72.

### Narrative Coding

Life narratives were audio recorded and transcribed verbatim. The first author, trained in a former study (Köber et al., 2015), divided life narratives into propositions, that is, into comprehensible main or subordinate clauses. Then, the first author trained four research assistants in applying the coding schemes of global narrative coherence, autobiographical reasoning, and self-event connections. Two research assistants coded autobiographical reasoning. The second author coded self-event connections in collaboration with the first author and, 6 months later, global narrative coherence with another research assistant. All research assistants were blind to the hypotheses of the study. To establish reliability between coders, two coders first independently coded 15 life narratives, which were then compared by means of Cohen’s kappa (κ) or intraclass correlation (*r*_IC_). Once the coders established substantial independent agreement (κ or *r*_IC_ > 0.70), both coders independently coded the remaining life narratives. All coder disagreements were resolved through consensus in discussion with the first author.

#### Global Coherence of Life Narratives

Three 7-point scales were used to rate the three kinds of global coherence of life narratives from the recipient’s point of view. The scale for temporal coherence measures how well the reader is temporally oriented. Lower values (1–2) were assigned when it remained unclear when and in what order life events occurred, whereas higher values (6–7) were given when the temporal order was consistently intelligible throughout the life narrative. Interrater reliability was *r*_IC_ = 0.99. The scale for causal–motivational coherence assesses how well a sense of the developmental significance of events is conveyed. Life narratives received lower values (1–2) when no development of the personality became clear and higher values (6–7) when the development of the personality became clear in its turning points and motives. Interrater reliability was *r*_IC_ = 0.98. The scale for thematic coherence measures how thematically coherent the life narrative is. Lower values (1–2) were assigned to life narratives when no or only one theme was addressed, whereas life narratives in which heterogeneous episodes or different areas of life (e.g., private and working life) were connected in a logical and comprehensible fashion received higher values (6–7). Interrater reliability was *r*_IC_ = 0.88.

#### Autobiographical Reasoning

Life narratives were coded for specific autobiographical arguments, constituting autobiographical reasoning. In line with prior research (Habermas and Köber, 2015a; Köber et al., 2015), we coded six autobiographical arguments: developmental status, biographical background, lesson learned, generalized insights, formative experience, turning points (see Table 1 for examples). Interrater reliability was good with Cohen’s κ = 0.79. In addition, we coded change-engendering self-event connections and stability-maintaining self-event connections. While change-engendering self-event connections explain perceived change in personality by specific life events, stability-maintaining self-event connections support stability of personality by describing an event or action as typical or, if contradicting personality, as atypical (Pasupathi et al., 2007). Interrater reliability was good with Cohen’s κ = 0.75.

**TABLE 1:** Excerpts of refugees’ life narratives with coding of autobiographical reasoning and self-event connections.

Autobiographical Arguments		
Developmental status	But in Africa it’s so difficult life, in Eritrea, when I was 16 years old the government of Eritrea used power or force to make me soldier, I said to him “Why? I am young ’til now, give me to, chance to learn or something.”	
Biographical background	I cannot forget my friends that dead in the uh Mediterranean Sea, because I can see by my eye, when they are crying, when they are, uh, they are sinking in the Mediterranean Sea and (-) that’s why I am always sad now but what can I do?	
Generalized insights	In my country there is no freedom, there is no, um, uh, human right, so you have to, you want to escape or you want to get out from the country because, uh, life is very short half my life is over, up to now not seeing the good life, that’s why I left my country, to find the good life.	
Lesson learned	I meet people where they are teaching me German language but could you say, uh, you have, uh, a close relation? You couldn’t say, I think. It is very difficult for me because there is, there are many things hidden (-) which they don’t want to say. So I realized what I need is not only German, but also to integrate and (-) making good relation with Germans.	
Formative experience	My parents were forced to move out of the house, My father and my mother were arguing about moving to another place, because that neighborhood was not well suited for a family, it’s like slums and there are a lot of other a, uh, other things that influence raising a child. So we moved to that place and yeah until I left Eritrea we lived there. So this event affected my life, I decided to uh to be careful about the situation, not to pollute myself, not to spoil myself by the surrounding. So I was like uh isolating myself from the neighborhood, uh because I felt that the area is not good for me.	
Turning points	Then my mother bec’, she became sick. She, she, she, suffered from cancer, yeah, lung cancer, yeah. And then I thought “No, she cannot die.” I stop everything, to work, to pray, I’m completely out of life, long time like this, I’m suffering with her.	

**Change-engendering self-event connections**		

Event explains change in personality	This is the first time me living alone, cooking for myself, cleaning up after myself and it’s a challenge, it’s a good, the first time I came here I couldn’t even cook an egg (-) I was, I was so lazy, my mother would cook for me, I didn’t have to do anything, I just had to go to school and (-) you know, living a normal kid’s life until all the situation that occurred (-) but even though, uhm, it has made, it has made me a better person I believe, living alone has taught me a lot of things.	
Event reveals unknown personality aspects	I wasn’t afraid of the situation what happened but I just, it just completely hit me that many people go through this everyday, and it’s not like an easy situation when you get arrested. There are also people being killed, you know, abducted in the middle of the night they would just break into your house and take the people. And at that moment there wasn’t fear inside me because I realized that I was fighting for some cause, you know. And at that moment I just realized that I was fighting for something and that I, that the society was with me and that was really empowering, you know, I was really empowered.	

***Self-event connections maintaining stability***		

Typical event explains personality	Like my father, he and I, we are lover of the land, and we be (-) when this this was really happiness for me and my life when we came back from Pakistan to our country, Afghanistan.	
Atypical event contradicts personality	I am, I am very strong, you know, I used to be very strong, so when I think that I beg here, that I, I don’t have any solution for my life, it’s disturbing me a lot, it’s not like me.	

**Self-event connections**		**Valence**

Personality dispositions	I have to be strong, disciplined, I have to be the role model for my younger brothers.	Neutral
Current values, beliefs about right or wrong	And once in my life I start again to fight for (–) fight against the government, and to bring peace, I was fighting for better life, to bring peace to my family and the world.	Negative
Current perspectives on the world or self	But I ask always in my mind, why don’t they [the German immigration office] call me to have some information and to have some interview, that’s why I am confused with my life and myself. Am I the person, (—) maybe it’s clear, the person who I am?	Negative
Personal growth	Then both of us uh decided to uhm getting divorce (-), and uh yeah, the year, uh, and, and after that I uh I tried to live uh, you know, by myself, independently from my parents or from anyone, I want to, I wanted to be good as a one person, not as a couple, and then I tried to build my life, you know, rebuild my life again.	Positive
Important relationships	I left my country with a lot of hopes, to have work, for a good life (–) for my family, with my wife, my sons, I love my sons, I love my wife.	Positive

#### Self-Event Connections

Following prior research (Merrill et al., 2016), an additional set of self-event connections were coded as any statements where participants explicitly linked a life event to their enduring sense of self. Capturing thus a rather stable view on the self, these coded self-event connections included the connection of life events to personality dispositions, current values, current perspectives about the world or self in general, personal growth, and important relationships informing the sense of self (see Table 1 for examples). Interrater reliability was moderate with Cohen’s κ = 0.70. Furthermore, these self-event connections were subcoded for valence, that is, as positive, negative, or neutral according to how the narrator appeared to view the connection in context (Banks and Salmon, 2013). As displayed in Table 1, self-event connections were coded as positive when the statement referred to a positive characteristic of the self, mentioned personal growth, or denoted a positive evaluation of the self. Self-event connections were coded as negative when the statement referenced a negative characteristic of the self or denoted a negative evaluation of the self. Self-event connections were coded as neutral when there was no evaluation of the self or the connection was not clearly positive or negative. Interrater reliability for the valence of self-event connection was very good with Cohen’s κ = 0.91.

## Results

### Preliminary Analyses

The number of all autobiographical arguments including change-engendering and stability-maintaining self-event connections was divided by the total number of propositions per life narrative, yielding the relative frequency of autobiographical arguments. Similarly, the relative frequency of all self-event connections and the proportion separated by their valence was calculated. Because of our directed hypotheses, the small sample size, and some non-normally distributed variables, one-tailed non-parametric correlations were calculated. More specifically, we relied on Kendall’s tau for testing our hypothesis because it is a rigorous estimate for smaller sample sizes (Howell, 1992). We first report the results on autobiographical meaning making and the sense of self-continuity (hypothesis 1) and second describe the findings on autobiographical meaning making and psychological distress (hypothesis 2).

### Autobiographical Meaning Making and the Sense of Self-Continuity

The sense of self-discontinuity tended to correlate with objective change in life (Kendall’s *τ* = 0.174, *p* < 0.10; Table 2), supporting the notion that biographical change disturbs the continuous sense of self. However, opposing hypothesis 1, the sense of self-discontinuity did not correlate with narrative coherence (hypothesis 1a), autobiographical reasoning (hypothesis 1b), or positive self-event connections (hypothesis 1c). Instead, objective change in life correlated negatively with global causal–motivational coherence of life narratives (Kendall’s *τ* = −0.241, *p* < 0.05; Table 2) and with negative self-event connections (Kendall’s *τ* = −0.275, *p* < 0.05; Table 2). This result indicates that the more life had changed, the less causal–motivationally coherent refugees depicted their life narratives and the more they narrated enduring aspects of the self in negative terms. This might hint at some difficulties to integrate the change that was caused by their flight into the broader autobiographical context. Those refugees who experienced greater changes in life since they fled their home country managed less well to explain how they had become the person they were at present.

**TABLE 2:** Kendall’s tau coefficients for relations between objective change in life, the sense of self-discontinuity, autobiographical meaning making, and psychological distress.

		Objective change in life	Sense of self-discontinuity	Temporal coherence	Causal– motivational coherence	Thematic coherence	Autobiographical reasoning	Self-event connections			
	
								Total	Positive	Negative	Neutral
Self-discontinuity	Objective change in life	—-	0.174^+^	–0.067	−0.241**	-0.140	-0.069	–0.126	0.192*	−0.275**	–0.063
	Sense of self-discontinuity	—-	—-	0.027	–0.007	0.021	-0.050	0.033	0.027	0.049	0.219^+^
Psychological distress	BSI–Global Severity Index	–0.052	0.261**	0.122	0.304**	0.185*	0.185*	0.063	0.009	0.059	0.407**
	IES–Intrusion	–0.026	0.404**	–0.029	0.122	-0.063	-0.126	–0.124	−0.209*	0.164	0.005
	IES–Avoidance	–0.124	0.241*	–0.005	–0.029	0.000	-0.204*	–0.045	−0.308**	0.129	0.183
	IES –Hyperarousal	–0.127	0.389**	0.077	0.127	0.068	-0.117	–0.058	−0.248**	0.168	0.221**

N = 31. BSI, Brief Symptom Inventory measuring general psychological distress; IES, Impact of Event Scale measuring specific (flight-related) psychological distress. **p (one-tailed) < 0.05, *p (one-tailed) < 0.10.

This result supports prior research that narrating change in life requires increased cognitive effort on autobiographical meaning making in order to render life stories coherent (Grysman and Hudson, 2010; Enz and Talarico, 2016). In other words, whether autobiographical meaning making is instigated to ensure the sense of self-continuity might depend on the amount of change in life. We therefore followed the procedure by Habermas and Köber (2015a) and divided our sample into two groups, thereby accounting for their differences in experienced objective change in life. We used the median of objective change in life (*Mdn* = 0.003) to split the participants in a group with less change in life (*N* = 16) and a group with more change in life (*N* = 15). Both groups did not differ significantly in their autobiographical meaning making or psychological distress (Table 3), as controlled for by non-parametric tests.

**TABLE 3:** Mean, standard deviation, and range of narrative and questionnaire measures.

	Total Sample (*N* = 31)				Group with less change in life (*N* = 16)				Group with more change in life (*N* = 15)			
	*M*	*SD*	Min.	Max.	*M*	*SD*	Min.	Max.	*M*	*SD*	Min.	Max.
	**Narrative Measures**											

Temporal coherence	4.23	1.73	1.00	7.00	4.50	1.93	1.00	7.00	3.93	1.49	1.00	6.00
Causal–motivational coherence	2.74	1.48	1.00	6.00	3.19	1.42	1.00	5.00	2.27	1.44	1.00	6.00
Thematic coherence	2.81	1.01	1.00	6.00	3.00	1.16	2.00	6.00	2.60	0.83	1.00	4.00
Autobiographical reasoning (relative frequency)	1.47	1.75	0.00	7.00	1.38	1.42	0.10	5.00	1.57	2.10	0.00	7.00
Self-event connections (relative frequency)	3.29	3.12	0.00	16.40	3.38	2.03	0.00	8.20	3.19	4.05	0.00	16.40
Positive (in% of total)	50.10	30.09	0.00	100.00	47.47	26.76	0.00	90.90	52.91	34.01	0.00	100.00
Negative (in% of total)	31.83	25.53	0.00	100.00	37.61	22.75	0.00	76.20	35.01	26.64	8.30	100.00
Neutral (in% of total)	8.39	12.64	0.00	50.00	8.68	13.42	0.00	50.00	8.07	12.21	0.00	33.30

	**Questionnaire Measures**											

Objective Change in Life (z-standardized)	–0.01	0.41	–0.78	0.78	–0.33	0.27	–0.78	0.01	0.33	0.21	0.03	0.78
Sense of self-discontinuity	3.66	1.28	1.50	6.00	3.52	1.41	1.50	6.00	3.82	1.16	1.75	5.50
BSI–Global Severity Index	0.77	0.58	0.00	2.56	0.77	0.57	0.00	1.83	0.77	0.61	0.04	2.56
IES–Intrusion	1.98	0.99	0.50	3.63	1.83	0.92	0.50	3.50	2.10	1.08	0.50	3.63
IES–Avoidance	1.78	0.95	0.14	3.71	1.85	1.14	0.14	3.71	1.73	0.81	0.63	3.25
IES–Hyperarousal	1.72	1.14	0.17	4.00	1.80	1.29	0.17	4.00	1.64	1.06	0.50	3.33

**FIGURE 1:**
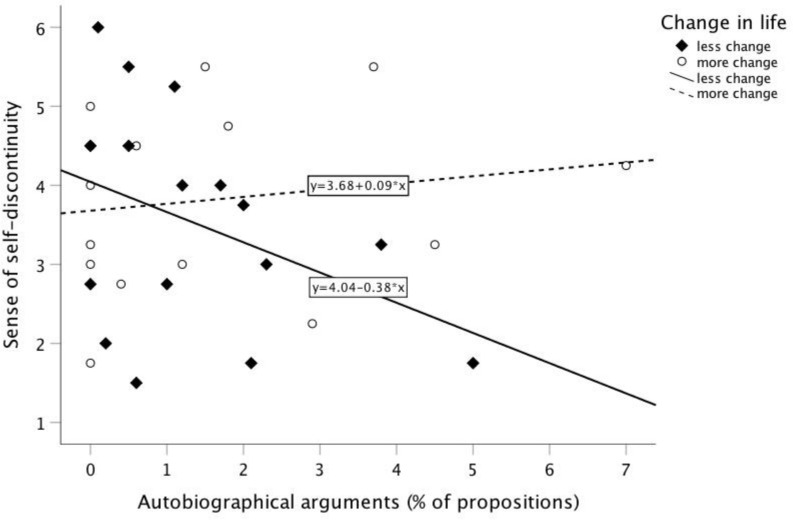
Scatterplot of proportion of autobiographical arguments in life narratives with sense of self-discontinuity by groups of objective change in life since displacement.

The correlations of the participants who experienced less change in life are displayed in Table 4. Objective change in life correlated positively with positive self-event connections (Kendall’s *τ* = 0.420, *p* < 0.05; Table 4). Apparently, the greater the change in life was since these participants left their home country, the more positive aspects they note in their personality, values, perspectives on the world, or relationships. However, neither the amount of positive self-event connection nor narrative coherence correlated significantly with the sense of self-discontinuity. Only autobiographical reasoning correlated negatively with the sense of self-discontinuity (Kendall’s *τ* = −0.358, *p* < 0.05; Table 4). Thus, in this subsample, with less experienced change in life since their flight, hypothesis 1b could be confirmed. Figure 1 shows the separate correlations of autobiographical reasoning with the sense of self-discontinuity for both subsamples. In the subsample with less experienced change in life since their flight, autobiographical reasoning appeared to buffer the sense of self-continuity after forced displacement (Table 4 and Figure 1). The predicted variance in this subsample was *R*^2^ = 0.15, indicating that the more autobiographical reasoning these participants displayed in their life narratives, the higher was their sense of self-continuity.

**TABLE 4:** Kendall’s tau coefficients for relations between objective change in life, the sense of self-discontinuity, autobiographical meaning making, and psychological distress for the group who experienced less objective change in life.

	Objective change in life	Sense of self-discontinuity	Temporal coherence	Causal–motivational coherence	Thematic coherence	Autobiographical reasoning	Self-event connections			
	
							Total	Positive	Negative	Neutral
Objective change in life	−	0.254	0.203	–0.045	0.080	0.017	0.025	0.420**	–0.185	–0.040
Sense of self-discontinuity	−	−	0.206	–0.222	0.192	−0.358*	–0.187	–0.085	–0.034	0.243
BSI–Global Severity Index	−0.050	0.322*	0.148	0.227	0.259	0.101	–0.008	–0.303	0.218	0.558**
IES–Intrusion	−0.333	0.205	0.000	0.290	0.348	–0.244	0.360	–0.296	0.225	0.119
IES–Avoidance	−0.250	0.442*	–0.108	0.049	0.305	–0.023	0.138	–0.395	0.138	0.464*
IES–Hyperarousal	−0.244	0.386	0.105	0.097	0.298	–0.067	0.449*	−0.568*	0.315	0.406

N = 16. BSI, Brief Symptom Inventory measuring general psychological distress; IES, Impact of Event Scale measuring specific (flight-related) psychological distress. **p (one-tailed) < 0.05. *p (one-tailed) < 0.10.

In contrast, in the subsample of participants who experienced more change in life since their flight, no significant correlation between the sense of self-discontinuity and autobiographical meaning making occurred (Table 5). Figure 1 shows that autobiographical reasoning in this subsample did not buffer the negative effect of their forced displacement on their sense of self-continuity. The predicted variance in this subsample was *R*^2^ = 0.03, remarkably lower than in the subsample who experienced less change in life.

**TABLE 5:** Kendall’s tau coefficients for relations between objective change in life, the sense of self-discontinuity, autobiographical meaning making, and psychological distress for the group that experienced more objective change in life.

	Objective change in life	Sense of self-discontinuity	Temporal coherence	Causal–motivational coherence	Thematic coherence	Autobiographical reasoning	Self-event connections			
	
							Total	Positive	Negative	Neutral
Objective change in life	−	0.107	0.213	–0.033	–0.295	–0.062	0.010	0.069	–0.188	–0.142
Sense of self-discontinuity	−	−	–0.185	0.302	–0.174	0.147	0.304	0.190	0.152	0.169
BSI–Global Severity Index	−0.153	0.156	0.182	0.364*	0.103	0.248	0.097	0.365*	–0.100	0.166
IES–Intrusion	−0.140	0.603**	–0.052	–0.017	−0.418*	–0.081	−0.406*	–0.127	0.110	–0.125
IES–Avoidance	−0.229	0.016	0.153	–0.224	–0.355	–0.240	–0.246	–0.250	0.078	–0.053
IES–Hyperarousal	−0.233	0.302	0.087	–0.035	–0.285	–0.211	−0.438*	0.000	–0.079	–0.018

N = 15. BSI, Brief Symptom Inventory measuring general psychological distress; IES, Impact of Event Scale measuring specific (flight-related) psychological distress. **p (one-tailed) < 0.05. *p (one-tailed) < 0.10.

This finding that only those refugees who experienced less change in life seemed to benefit from autobiographical meaning making is surprising because it contradicts prior research (Habermas and Köber, 2015a; Vanderveren et al., 2020). Therefore, we additionally explored whether the time that had passed since they fled their home country or the length of their stay in Germany would shed light on the relation between autobiographical meaning making and the sense of self-discontinuity. However, in both subsamples, the sense of self-discontinuity did not correlate significantly with the time that had passed since the flight or with the length of their stay in Germany. The group with more change in life showed merely a positive correlation between neutral self-event connections (Kendall’s *τ* = 0.379, *p* < 0.05) and the time passed since the flight.

### Autobiographical Meaning Making and Psychological Distress

Regarding hypothesis 2, correlational analyses in the entire sample showed that the measures of general (BSI) and specific psychological distress (IES-R) did not correlate significantly with objective change in life (Table 2). However, the more participants felt psychologically distressed, the greater was their sense of self-discontinuity. Especially, intrusive memories and emotional hyperarousal related to an increased sense of self-discontinuity (Table 2). Counter to hypothesis 2, general psychological distress correlated positively with causal–motivational (Kendall’s *τ* = 0.304, *p* < 0.05; Table 2) and by trend with thematic narrative coherence (Kendall’s *τ* = 0.185, *p* < 0.10; Table 2), autobiographical reasoning (Kendall’s *τ* = 0.185, *p* < 0.10; Table 2), and positive self-event connections (Kendall’s *τ* = 0.407, *p* < 0.05; Table 2). Moreover, less positive self-event connections went along with greater symptoms of specific flight-related stress such as with greater memory intrusion (Kendall’s *τ* = −0.209, *p* < 0.10; Table 2), avoidance of the stressing event (Kendall’s *τ* = −0.308, *p* < 0.05; Table 2), and emotional hyperarousal (Kendall’s *τ* = −0.248, *p* < 0.05; Table 2). Last, emotional hyperarousal correlated positively with neutral self-event connections (Kendall’s *τ* = 0.221, *p* < 0.05; Table 2). Altogether, greater autobiographical meaning making appeared to worsen the psychological stress in the refugees, or greater psychological stress in the refugees reduced the capacity for autobiographical meaning making, especially for positive self-event connections.

Looking at the separate subsamples confirms these results and adds different nuances. While the group with less change in life also showed by trend a greater sense of self-discontinuity in relation to greater general psychological distress (Kendall’s *τ* = 0.322, *p* < 0.10; Table 4) and avoidance (Kendall’s *τ* = 0.442, *p* < 0.10; Table 4), autobiographical meaning making barely correlated with psychological distress (Table 4). Only neutral self-event connection correlated positively with general psychological distress (Kendall’s *τ* = 0.559, *p* < 0.05; Table 4) and by trend with avoidance (Kendall’s *τ* = 0.464, *p* < 0.10; Table 4). Furthermore, emotional hyperarousal tended to correlate positively with the total amount of self-event connections (Kendall’s *τ* = 0.449, *p* < 0.10; Table 4) but negatively with positive self-event connections (Kendall’s *τ* = −0.568, *p* < 0.10; Table 4). Overall, these results indicate that in the subsample with less change in life, general psychological distress and avoidance of the stressor tolerate only neutral self-event connections or *vice versa*. Further, hyperarousal and positive self-event connections tend to preclude each other.

The group with more change in life showed a positive correlation of the sense of self-discontinuity and intrusion (Kendall’s *τ* = 0.603, *p* < 0.05; Table 5), indicating that intrusive thoughts and the sense of discontinuity maintain each other in this subsample. Moreover, greater psychological distress tended to correlate positively with greater causal–motivational coherence (Kendall’s *τ* = 0.364, *p* < 0.10; Table 5) and more positive self-event connections (Kendall’s *τ* = 0.365, *p* < 0.10; Table 5). Greater intrusion, however, tended to correlate negatively with thematic coherence (Kendall’s *τ* = −0.418, *p* < 0.10; Table 5) and the total amount of self-event connections (Kendall’s *τ* = −0.406, *p* < 0.10; Table 5). Emotional hyperarousal tended to correlate negatively with the total amount of self-event connections (Kendall’s *τ* = −0.438, *p* < 0.10; Table 5). Again, these results indicate that autobiographical meaning making and psychological distress tend to mutually impair each other, although less intrusion and less hyperarousal tend to facilitate greater autobiographical meaning making, or autobiographical meaning helps to reduce intrusion and hyperarousal.

## Discussion

Inspired by prior research (Habermas and Köber, 2015a), we here examined whether autobiographical meaning making buffers the impact of extreme biographical disruptions such as forced displacement on the sense of self-discontinuity and psychological distress. Overall, we found a buffering effect only in refugees who experienced less objective change in life since their forced migration and an overall trend of autobiographical meaning and psychological distress to be detrimental to each other. Below we discuss the main results as well as the limitations of the study.

As evidenced, the more participants felt psychologically distressed, the greater was their sense of self-discontinuity (Table 2). This confirms that psychological distress and the sense of selfhood are interconnected, probably mutually impairing each other once either psychological well-being or the sense of self-continuity is disturbed (Chandler et al., 2003; Rzesnitzek, 2014). The greater sense of self-discontinuity was specifically related to greater frequencies of intrusive memories and emotional hyperarousal, corroborating the crucial role of autobiographical memory for the continuous sense of self (Conway et al., 2004; Bluck and Liao, 2013) but also for the maintenance of trauma-related symptoms (Berntsen et al., 2003; Berntsen, 2009). Yet, results for the entire group showed no alleviating effect of autobiographical meaning making on the sense of self-discontinuity or psychological distress. In contrast, we found that the more objective change in life had happened in the participants’ lives since their displacement, the less causal–motivational coherent were their life narratives (Table 2). Furthermore, those with greater general psychological distress tended to engage in more autobiographical meaning making, displayed in greater causal–motivational and thematic coherence, more autobiographical reasoning, and more neutral self-event connections. On the other hand, greater specific flight-related psychological stress and a lack of positive self-event connections tended to mutually sustain each other (Table 2), supporting prior research (Banks and Salmon, 2013). Altogether, these results show that refugees were actively trying to cope in their life narratives with their current psychological stress, their past, and the experienced change in life, but the autobiographical meaning making did not yield a coherent narrative account that provides a continuous sense of selfhood but instead worsens the psychological distress. One possible reason is that participants’ meaning making attempts did not succeed in meaning made, which would sufficiently reconcile their past with the current life (Park, 2010; Huang et al., in press) but that their autobiographical meaning making resembles rather rumination maintaining the current psychological distress (Lilgendahl et al., 2013; Sales et al., 2013).

Another factor is certainly the extreme stress and the extreme change in life that refugees experience. Even though prior research evidences that autobiographical meaning making relates to a higher sense of self-continuity and well-being when coping with negative life events (Banks and Salmon, 2013; Habermas and Köber, 2015a; Merrill et al., 2016; Huang et al., in press; Vanderveren et al., 2020), in none of these studies individuals experienced a change in their physical and sociocultural environment as fundamental as most refugees have. While all biographical disruptions and specific traumatic events are challenging for the sense of self and psychological well-being, it is rare that the relative stability of one’s physical environment, social relations, and routine activities are threatened simultaneously. Forced migration, however, involves traumatizing experiences encountered in the refugees’ home countries and during displacement, causes the loss of the physical and social environment, often endangers bodily safety, and demands the integration into an unknown society often under arduous bureaucratic, economic, and societal circumstances (Porter and Haslam, 2005; Ullmann et al., 2015; James et al., 2019). Thus, forced migration entails several biographical disruptions that accumulate and protract over the periods of preflight, flight, exile, and resettlement, which can last several years.

This aspect of protracted stress after the flight was measured in this study by assessing the objective change in life that happened since the forced displacement. Remarkably, we found beneficial effects of autobiographical meaning making on the sense of self-continuity only in those refugees who had experienced less change in their lives since their flight. Because this group had to cope with less postdisplacement stress, it might have been easier for them to cope narratively with their forced displacement and their current present. The significant correlation between objective change in life and the positive self-event connections (Table 4) suggests that these refugees managed to maintain a positive self-concept amid the change or that the change in life revealed positive self-aspects to them. This positive view on the self seems to reduce the emotional hyperarousal, while autobiographical reasoning preserves the sense of self-continuity (Table 4). These results seem further supported by our finding that these effects were unrelated to the time gone by since displacement or the time since their arrival in Germany. Sheer passing of time did not affect the sense of self-continuity. Rather, if the change in life was not overwhelming, individuals seemed to be able to extract autobiographical meaning from extreme biographical disruptions, buffering their continuous sense of self and alleviating psychological distress.

For those, however, who experienced greater change in life in addition to and since their forced displacement, autobiographical meaning making appeared to be detrimental for the continuous sense of self and psychological well-being. Very likely, the several other biographical disruptions such as losses of significant others, severe health issues, and moves within Germany that this subsample experienced in addition to their forced migration nullify the compensating effect of autobiographical meaning making on the continuous sense of self. Accordingly, we found causal–motivational coherence and positive self-event connections to tend to positively correlate with psychological distress (Table 5). The total amount of self-event connections, however, and thematic coherence were by trend negatively correlated to memory intrusion and emotional hyperarousal (Table 5). In line with other research, these results imply that autobiographical meaning making is more easily possible and beneficial if the memory load is emotionally bearable (Rubin et al., 2008). As long as pre- and/or postdisplacement stressors maintain psychological distress, the avoidance to remember and repress autobiographical meaning making can be a strategy to isolate painful memories in order to continue life and plan the future amid ongoing stressors (Gemignani, 2011).

Obviously, this study shows several limitations. First of all, we managed to recruit only a small number of participants because many were unwilling to talk about their lives and others were afraid of endangering their asylum claims in Germany when telling us their life stories. Second, it was impossible for us to determine the psychological distress and potential trauma the participants experienced prior to fleeing their country or individual psychological resources such as resilience. Thus, we could not determine the individual vulnerability to develop mental illnesses due to the trauma and extreme stress they go through (Ullmann et al., 2015; Wildschut et al., 2019). Third, although all participants were sufficiently fluent to fulfill the requirements of data collection, testing them in their native language might have put the participants more at ease when telling their lives.

Nevertheless, our results replicate and extend prior research indicating that autobiographical is a potential means to explain and cope with biographical disruptions, thereby preserving the sense of self- continuity and alleviating psychological distress. However, we also found the nuance that if biographical change is too overwhelming or still ongoing, or the memory is load emotionally unbearable, autobiographical meaning making might be detrimental for the sense of self-continuity and psychological well-being. This points to both situational factors and individual differences influencing the benefit of autobiographical meaning for the sense of selfhood. The individual threshold of how much change a person can cope with and to what extent it needs narrative integration of biographical disruptions to make the future livable remains thus to future research.

## Data Availability Statement

The datasets presented in this article are not readily available because the data are highly confidential. Sharing of the data is not entitled as per the ethical approval. Requests to access the numerical and anonymized datasets should be directed to CC, Christin.Camia@zu.ac.ae.

## Ethics Statement

The studies involving human participants were reviewed and approved by Ethikkommission des Fachbereichs 5: Psychologie und Sportwissenschaften Goethe University Frankfurt/Main, Germany # 2015-127. The patients/participants provided their written informed consent to participate in this study.

## Author Contributions

CC designed the study, conducted some of the interviews, supervised the coding, analyzed the data, and drafted the manuscript. RZ completed two coding schemes and gave comments on earlier versions of this manuscript. Both authors contributed to the article and approved the submitted version.

## Conflict of Interest

The authors declare that the research was conducted in the absence of any commercial or financial relationships that could be construed as a potential conflict of interest. The handling editor declared a past co-authorship with one of the authors CC.
